# Rehabilitation of an existing device implant pocket using a biologic extracellular matrix envelope

**DOI:** 10.1016/j.hrcr.2023.08.007

**Published:** 2023-08-22

**Authors:** Ankur Srivastava, Hemal M. Nayak

**Affiliations:** Center for Arrhythmia Care, The University of Chicago Medicine, Chicago, Illinois

**Keywords:** Cardiac implantable electronic device (CIED), CIED envelope, Foreign body reaction, Capsulectomy, Partial capsulectomy, Pacemaker, Implantable cardioverter-defibrillator (ICD), Fibrosis, Electrophysiology, Histologic analysis

**Key Teaching Points**

• Implantation of a cardiac implantable electronic device often leads to fibrous encapsulation of the device and/or leads, which may increase reoperative difficulty and complication risk. Techniques that mitigate fibrotic tissue formation may facilitate reoperative procedures and improve clinical outcomes.
• We present the first histologic evidence that placing a biologic device envelope within an existing densely fibrotic capsule can rehabilitate the device pocket by forming a new layer of cellular and vascular tissue surrounding the device.
• The results from this case report suggest there may be potential regenerative benefits of using a biologic extracellular matrix envelope during reoperative procedures; however, larger studies will allow further investigation.

## Introduction

Cardiac implantable electronic device (CIED) implantation comes with a risk of hypovascular fibrosis from a foreign body reaction to the generator and/or leads.^1^ Biologic extracellular matrix (ECM) CIED envelopes have been shown to alter the foreign body reaction and lead to significantly less fibrous lead entrapment, easier generator and lead mobilization, less procedural difficulty, and thinner mature tissue capsules at reoperation compared to using no envelope at initial implantation.^2^ However, limited clinical experience exists on the outcome of implanting biologic envelopes within existing fibrotic capsules. We present a unique case that highlights our findings from placing a CIED within a biologic ECM envelope and implanting it within an existing fibrotic CIED capsule during a revision procedure.

## Case report

**Figure 1:**
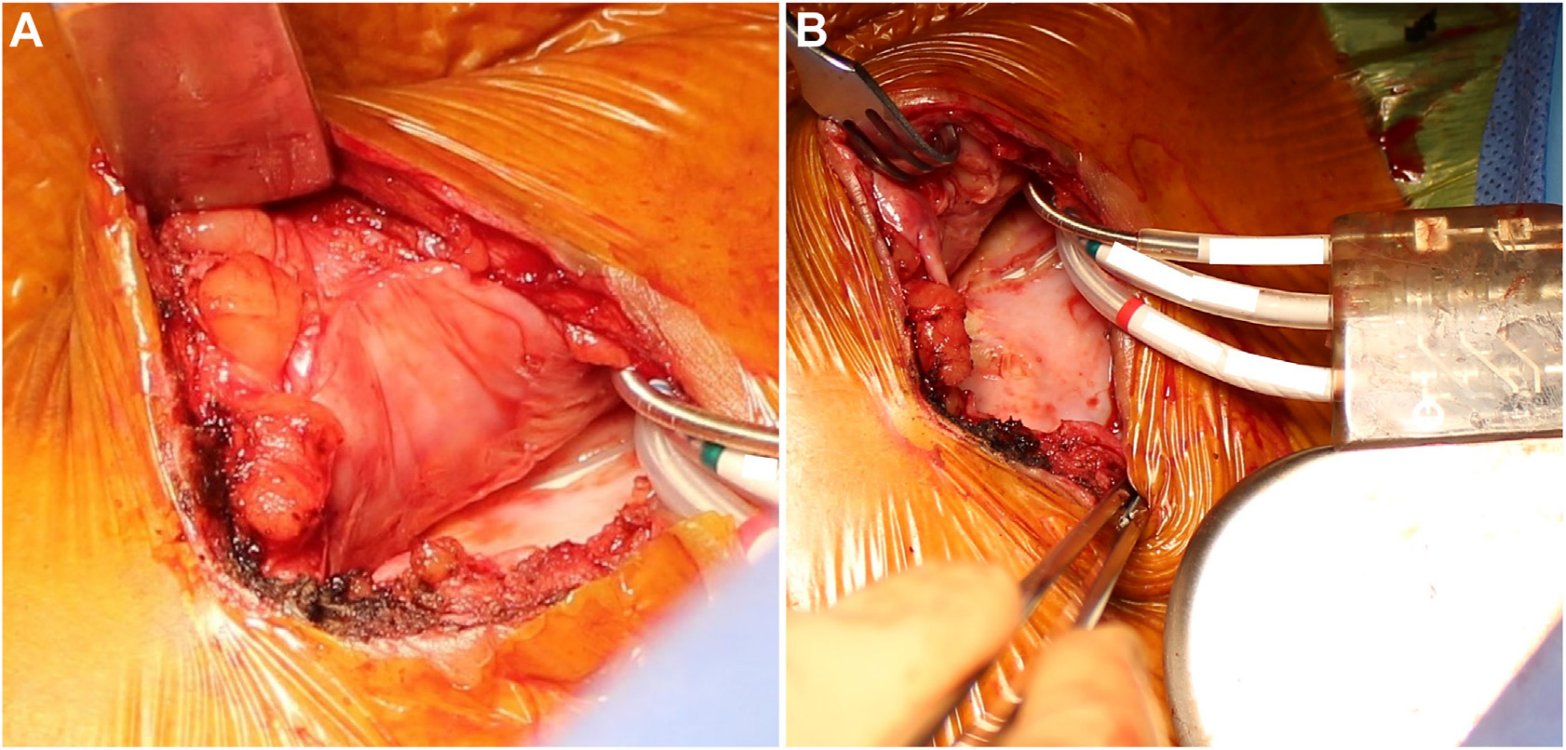
**A:** During reoperation 5 months after the initial reoperative procedure, gross inspection of the reoperative pocket demonstrated a thin, vascular pocket lining within the previous fibrotic capsule. **B:** Inspection revealed no fibrosis or adhesions present on the generator or leads.

A 73-year-old immunocompetent male patient with no renal issues received an implantable cardioverter-defibrillator during a change-out reoperative procedure in 2020, which included partial capsulectomy of the existing fibrotic pocket to remove portions of the capsule necessary to free the device and leads. At the time of surgery, he was not taking any antiplatelets or anticoagulants, aside from 325 mg aspirin daily. The patient received intravenous vancomycin prophylaxis 1 hour prior to the procedure, the skin was prepared with chlorhexidine, and the wound was irrigated with antibiotic. To rehabilitate the existing fibrotic pocket, the new implantable cardioverter-defibrillator was first placed into a biologic envelope derived from porcine small intestine submucosa ECM (CanGaroo® Envelope size Large; Aziyo Biologics Inc, Silver Spring, MD), then implanted. Five months postprocedure, the patient experienced lead-related complications that resulted in the need for an additional reoperation. When the device pocket was reopened, a thin, soft vascularized capsule was visible lining the entire pocket, the generator and leads were free of fibrosis and calcification, and there was no gross visible remnant of the envelope (Figure 1a and 1b).

**Figure 2:**
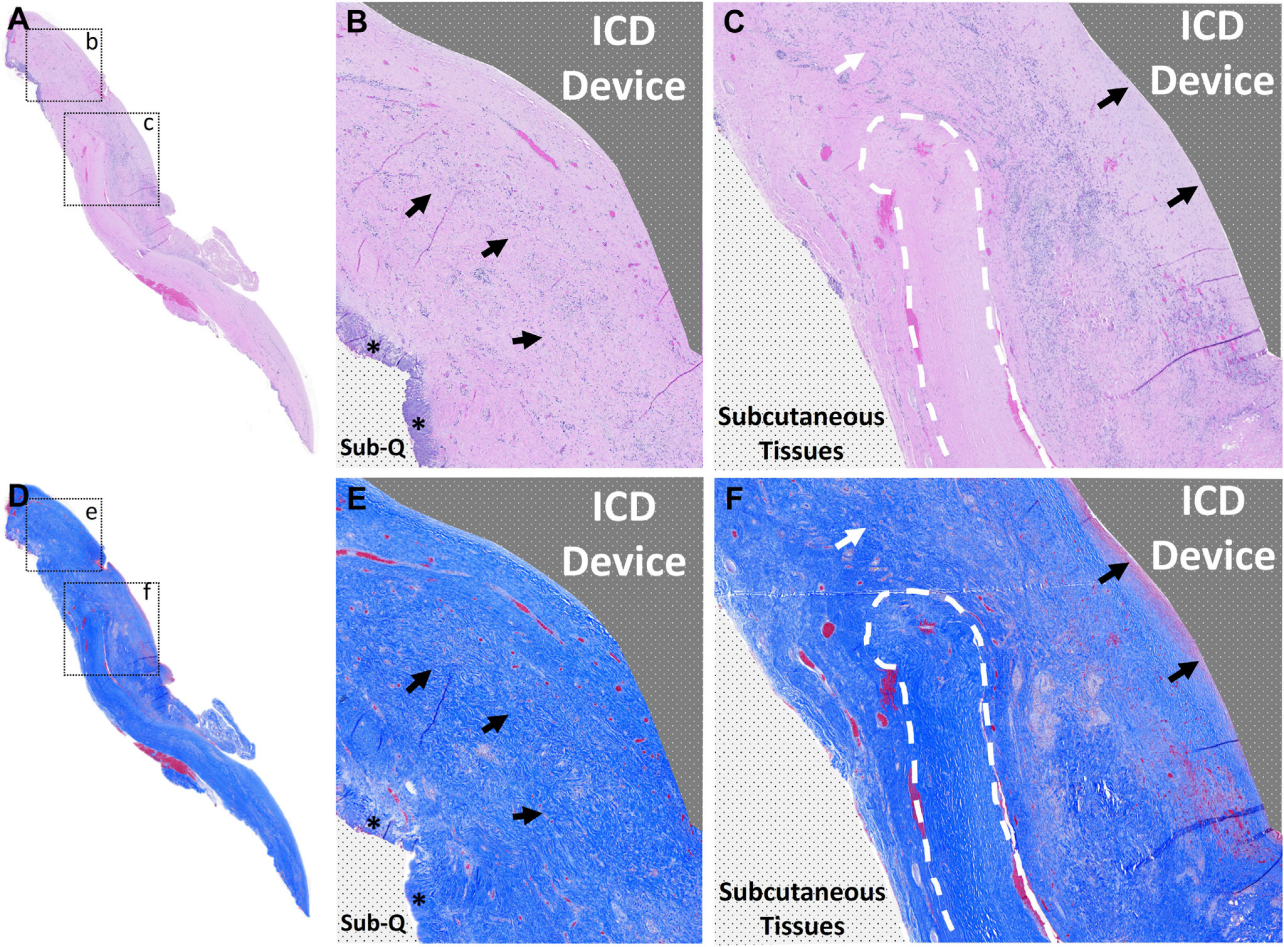
Full-field view of (**a**) hematoxylin-eosin and (**d**) Masson’s trichrome staining of a full-thickness sample taken from the reoperative pocket. Areas of interest are marked in black dashed boxes. **b, e:** Histologic findings in these areas of interest exhibited an area that previously underwent capsulectomy with evidence of a new cellular and vascular tissue layer (*arrows*) in continuous contact with chest wall subcutaneous (Sub-Q) tissue (*asterisks*) (10× magnification). **c, f:** Inside an adjacent sample including a remnant of the prior fibrotic capsule (*white dashes*), the new cellular and vascular layer is in continuity with surrounding subcutaneous tissues where capsulectomy was performed (*white arrow*), and a thin layer of organized collagen fibers form the new capsule lining (*black arrows*), which was adjacent to the implantable cardioverter-defibrillator (10× magnification). ICD = implantable cardioverter-defibrillator.

A full-thickness anterior pocket wall biopsy was taken and sent for independent histopathology. Hematoxylin-eosin and Masson’s trichrome staining confirmed no active inflammation and a new pocket wall consisting of cellular and vascular tissue with a thin collagenous lining, which was consistent with fully remodeled ECM envelope. No remnants of the previously implanted envelope were apparent in this sample. Where previous capsulectomy had been performed (Figure 2b and 2e), the new cellular and vascular pocket wall (arrows) was continuous with chest wall subcutaneous tissues (asterisks). Adjacent to that area within prior capsule remnant (where capsulectomy had not been performed) (Figure 2c and 2f, white dashes), there was a new cellular and vascular layer replacing the pocket lining (black arrows), which was in continuity with the surrounding subcutaneous tissues where capsulectomy had been performed (white arrow). The patient had an uneventful recovery and is still stable at 3 years postoperation.

## Discussion

This unique case provided an opportunity to evaluate the outcome of using a biologic ECM envelope within an existing fibrotic CIED device pocket. Implanted CIEDs frequently stimulate a foreign body reaction within the host, leading to hypovascular fibrous capsule development surrounding the device and/or leads, potentially increasing reoperative difficulty and risk of major complications such as infection.^1^ The observed increase in infection risk during reoperative procedures has been hypothesized to be due to the fibrous capsule acting as a nidus for infection by providing a protective barrier surrounding bacterial colonization on the device (introduced during the previous implantation procedure) from circulating immune cells until the capsule is disrupted.^3^ Patients expected to undergo multiple generator changes, upgrades, or revisions throughout life are especially impacted, as the risk of complications such as infection and hematoma increases with each additional procedure.^4^ Techniques that may mitigate the formation and/or persistence of dense fibrotic capsule tissue across procedures may alleviate this heightened risk of complications.

Biologic ECM scaffolds are well known for their tissue remodeling properties, which are due to intrinsic bioactive factors that recruit stem cells, promote angiogenesis, and modulate the host inflammatory response.^5^ Only naturally derived biologic materials, such as small intestine submucosa ECM, contain these regenerative molecules. In a recent preclinical study, animals with implanted biologic ECM envelopes encasing CIEDs had greater device stabilization and more vascularized capsules compared to implanting the device alone.^6^ These results also translate to the clinic: interim results from the HEAL Study (ClinicalTrials.gov #NCT04645173) indicate that the use of biologic ECM envelopes during initial device implantation can significantly reduce fibrous lead entrapment and simplify reoperative procedures by facilitating generator and lead mobilization, decreasing procedural difficulty, and decreasing the need for capsulectomy by >73% compared to implanting the device alone.^2^ However, the outcome of implanting a biologic envelope within an existing fibrotic capsule was previously undocumented.

This case report highlights the potential benefits of using a biologic device envelope during reoperative procedures. Histology confirmed that the biologic envelope had fully resorbed within the 5-month implantation period and was replaced by a thin layer of cellular and vascular host tissue, which simplified the subsequent reoperation by facilitating generator and lead mobilization, reducing procedural difficulty, and eliminating the need for any capsulectomy. Partial capsulectomy was performed during the first reoperative procedure, which may have supported the rapid transformation of the biologic envelope into the new cellular and vascular pocket tissue that we observed during the subsequent reoperation. Perhaps if no capsulectomy is performed in a fibrotic pocket prior to placement of a biologic envelope, the incisional site may still provide a sufficient host–scaffold interface and could result in the same outcomes, albeit over a slower period. However, further studies are needed to validate that hypothesis. Our results suggest that partial capsulectomy is sufficient to provide a productive interface for rapid tissue remodeling to occur, which lessens the risk of complications compared to performing full capsulectomy.^7^^,^^8^

## Conclusion

Our findings provide the first histologic evidence that placing a biologic device envelope within an existing densely fibrotic capsule (with partial capsulectomy) can create a new cellular and vascular tissue layer within the pocket for the implanted device. When used in this manner, biologic envelopes may potentially mitigate some of the issues associated with reusing fibrotic implant pockets at device reoperation, potentially simplifying reoperations and decreasing complications, therefore improving long-term implant site health for patients. Additional clinical investigations are warranted to further explore the biological benefits of biologic device envelopes.
